# Successful Treatment of Very Severe Sinusoidal Obstruction Syndrome After Gemtuzumab Ozogamicin With Transjugular Intrahepatic Portosystemic Shunt, Defibrotide, and High-Dose Corticosteroids: A Case Report

**DOI:** 10.7759/cureus.67682

**Published:** 2024-08-24

**Authors:** Naomi Thielemans, Nathan De Beule, Frans Van den Bergh, Pierre Lefesvre, Ann De Becker

**Affiliations:** 1 Department of Clinical Hematology, Universitair Ziekenhuis Brussel, Brussels, BEL; 2 Department of Interventional Radiology, Universitair Ziekenhuis Brussel, Brussels, BEL; 3 Department of Anatomopathology, Universitair Ziekenhuis Brussel, Brussels, BEL

**Keywords:** gemtuzumab ozogamicin, acute myeloid leukemia, tips, vod, case report

## Abstract

Sinusoidal obstruction syndrome (SOS) is a rare but potentially life-threatening complication, usually described in the setting of hematopoietic stem cell transplantation (HSCT). The very severe forms have a high mortality rate (>80%) and need fast recognition and urgent treatment. In this case report, we describe a unique and successful treatment strategy. We present a 27-year-old patient with newly diagnosed CD33+ acute myeloid leukemia (AML). She was treated with induction chemotherapy (7+3 regimen) and gemtuzumab ozogamicin (GO). In the absence of other major risk factors, she developed a very severe SOS with multiple organ failure. She was successfully treated with the urgent insertion of a transjugular intrahepatic portosystemic shunt (TIPS), defibrotide, and high-dose corticosteroids. This case of successful treatment for very severe SOS supports a combination strategy involving the immediate mechanical reduction of portal hypertension through TIPS and drug-mediated inhibition of microvascular thrombosis. Furthermore, this case shows the need for an improved prevention strategy, including the identification of additional risk factors and biomarkers.

## Introduction

Acute myeloid leukemia (AML) is a highly aggressive malignancy. While classical chemotherapy remains the cornerstone of treatment, advances in understanding the disease’s pathogenesis have led to the development of targeted therapies, such as gemtuzumab ozogamicin (GO). However, these new treatments can come with specific side effects, leading to new challenges.

Sinusoidal obstruction syndrome (SOS), also known as veno-occlusive disease of the liver, is a serious and potentially fatal complication that predominantly occurs in the context of hematopoietic stem cell transplantation (HSCT). While traditionally associated with HSCT, SOS can also develop following treatment with chemotherapeutic agents or immunotherapies, such as GO [1]. This condition is marked by damage to the liver’s small blood vessels, leading to portal hypertension, liver dysfunction, multi-organ failure, and even death in severe cases. Despite advances in prevention and treatment, the management of very severe SOS remains challenging.

This report discusses a case of very severe SOS in a young patient without comorbidities who was treated with GO for AML. It highlights the complexities of managing this life-threatening condition and emphasizes the importance of early recognition and intervention.

## Case presentation

We present a 27-year-old female patient with newly diagnosed CD33+ AML. She was started on induction chemotherapy with daunorubicin and cytarabine. Next-generation sequencing and cytogenetics were not yet available and thus GO was added to the treatment regimen on days 1, 4, and 7.

**Figure 1:**
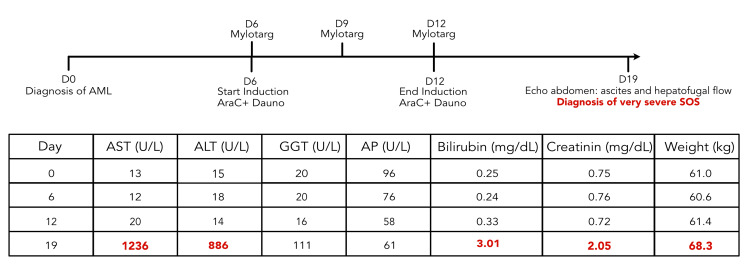
Timeline from diagnosis to development of SOS with corresponding lab results SOS: sinusoidal obstruction syndrome; AML: acute myeloid leukemia; AST: aspartate aminotransferase; ALT: alanine aminotransferase; GGT: gamma-glutamyl transferase; AP: alkaline phosphatase

Six days after completion of induction, the patient developed abdominal pain with a distended abdomen and a more than 10% weight increase. Laboratory results showed renal failure and markedly elevated liver function tests including bilirubin (Figure 1). Abdominal ultrasound revealed ascites and hepatofugal flow in the absence of macroscopic venous thrombosis. A clinical diagnosis of very severe SOS was made based on the revised criteria of the European Society for Blood and Marrow Transplantation (EBMT) [2].

**Figure 2:**
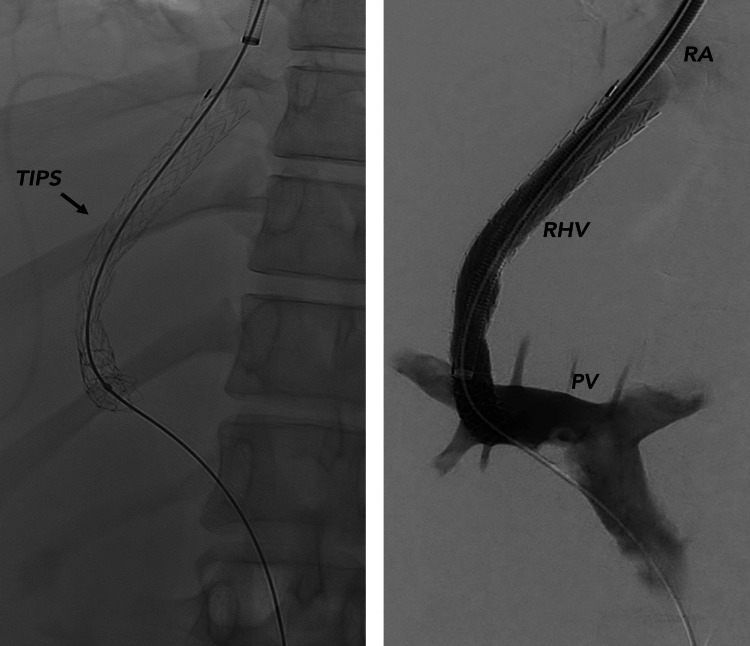
TIPS: Pressure gradient (RA-VP) drops from 25 mmHg to 10 mmHg after TIPS procedure PV: portal vein; RA: right atrium; RHV: right hepatic vein; TIPS: transjugular intrahepatic portosystemic shunt

The patient was transferred to the intensive care unit and treated urgently with a TIPS to reduce portal hypertension (Figure 2).

**Figure 3:**
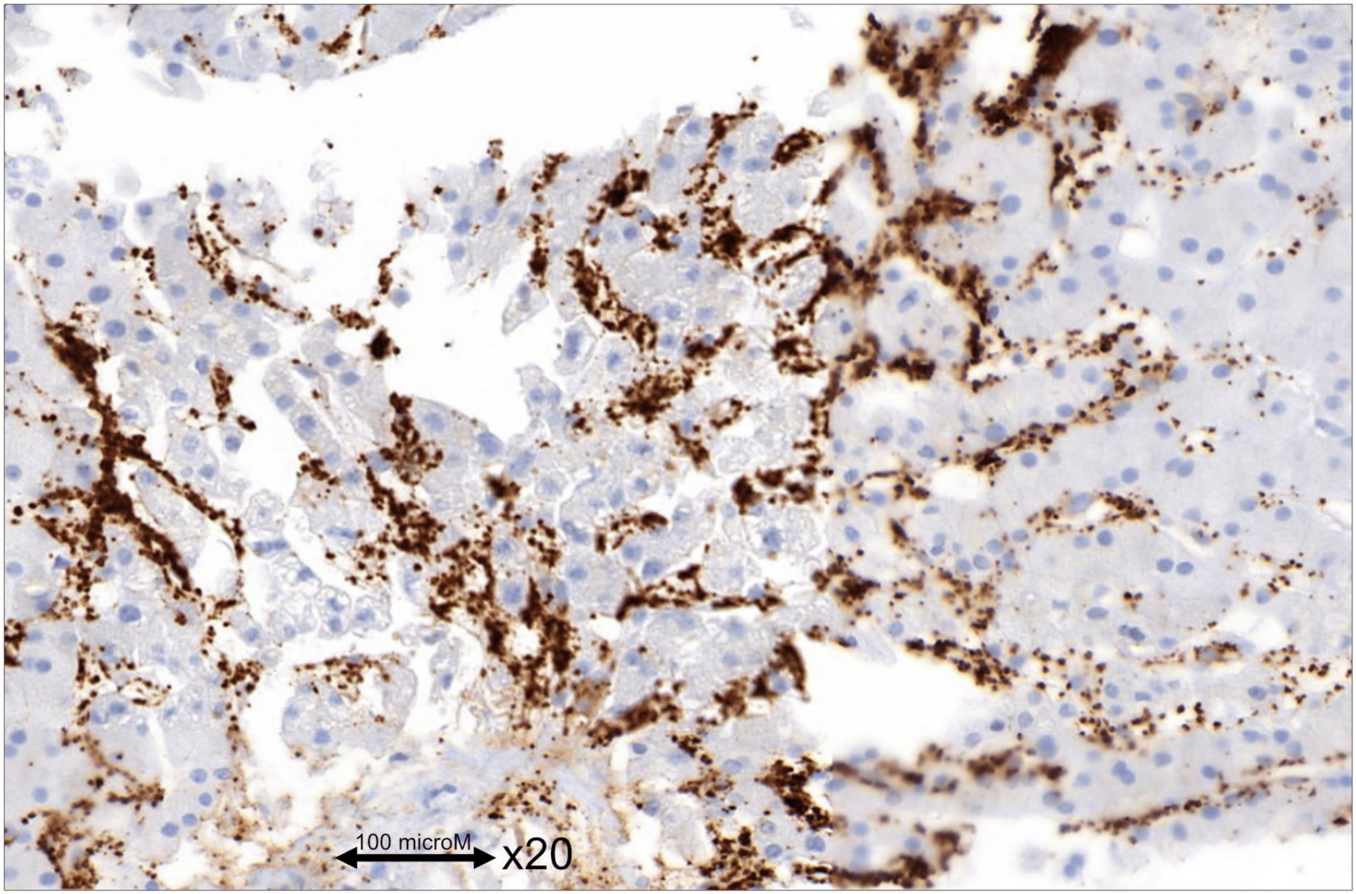
Histopathology of liver biopsy The liver sinus is clumped with thrombi. CD61 stains the platelets in hepatic capillaries.

This resulted in a drop in hepatic venous pressure gradient and rapid improvement of ascites and renal function. Concurrent liver biopsy confirmed SOS diagnosis (Figure 3).

**Figure 4:**
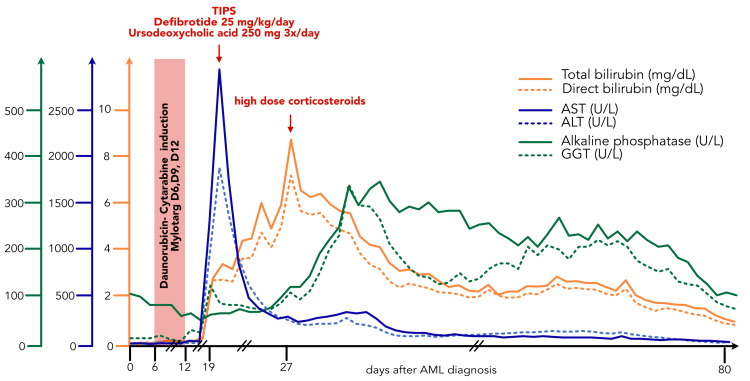
Evolution of liver enzymes over time Red arrows show different treatment regimens. TIPS: transjugular intrahepatic portosystemic shunt; AST: aspartate aminotransferase; ALT: alanine aminotransferase; GGT: gamma-glutamyl transferase; AML: acute myeloid leukemia

Subsequently, treatment with defibrotide 25 mg/kg/day and ursodeoxycholic acid 250 mg 3 tid was started. In the following days, a quick improvement of the abdominal symptoms and marked reduction of hepatocellular enzymes was observed. Unfortunately, this was offset after a few days by a progressive increase in cholestatic liver function tests despite confirmation of a correct TIPS position. We interpreted this as a signal that the SOS was not well controlled. Eight days after the TIPS procedure, high-dose corticosteroids were started with pulse doses of 500 mg methylprednisolone IV once daily for three days, followed by tapering [3]. This resulted in a prompt reduction of cholestatic liver tests. The patient was able to leave the ICU after three weeks. Liver function tests continued to improve and returned to normal two months after SOS diagnosis (Figure 4).

After a rehabilitation course, the patient was able to successfully resume chemotherapy with the omission of GO.

## Discussion

The diagnosis of SOS is based on diagnostic criteria, which were recently revised by the EBMT and distinguish pediatric from adult patients (Table 1) [2].

**Table 1:** Diagnostic criteria of sinusoidal obstruction syndrome in adults proposed by the expert panel of the European Society for Blood and Marrow Transplantation in 2023 Symptom onset: <21 days after HSCT: classic SOS; >21 days after HSCT: late SOS In each patient, symptoms/signs cannot be attributed to other causes. HVPG: hepatic venous pressure gradient; SOS: sinusoidal obstruction syndrome

Probable	Clinical	Proven
≥2 of the following criteria:	Bilirubin ≥ 2 mg/dl and ≥2 of the following criteria:	≥1 of the following criteria:
Bilirubin ≥ 2 mg/dl	Painful hepatomegaly	Histologically proven SOS
Painful hepatomegaly	Weight gain > 5%	Hemodynamically proven SOS (HVPG ≥ 10 mmHg)
Weight gain > 5%	Ascites	
Ascites		
US/elastography suggestive of SOS		

Additionally, the EMBT criteria define "mild," "moderate," "severe," and "very severe" SOS based on time of onset, bilirubin concentration, kinetics of hyperbilirubinemia, degree of transaminase elevation, weight gain, and renal function. According to this grading system, our patient developed a very severe SOS.

Different risk factors are associated with the development of SOS after HSCT, including patient-related factors (such as age), hepatic risk factors, and transplant/conditioning regimen-related factors [4]. These risk factors can be classified as major and minor. Among the major risk factors, treatment with anti-leukemic immunoconjugates, particularly GO, is now recognized as a significant contributor. GO is an anti-CD33 antibody-drug conjugate, reimbursed for patients with newly diagnosed CD33+ AML who have a good or intermediate risk according to 2022 ELN risk stratification. The drug first received FDA approval in 2000 as monotherapy for elderly patients with AML, who were unfit for traditional chemotherapy. However, this approval was quickly withdrawn due to safety concerns, particularly related to its potential for causing liver toxicity and SOS. Additionally, clinical studies at that time failed to demonstrate a clear clinical benefit, further contributing to the withdrawal. Despite these early setbacks, GO was re-approved in 2017 after new data emerged, which highlighted its efficacy in patients with good or intermediate risk when administered in lower, fractionated doses [5].

While SOS is primarily associated with HSCT and HSCT is often included in the diagnostic criteria, studies have shown that it can also develop in patients treated with high-dose chemotherapy or anti-leukemic immunoconjugates like GO without HSCT. The incidence of SOS in patients treated with GO alone is reported to be up to 10%, and up to 40% when GO is administered within three months of an allogeneic HSCT [1]. However, more recent analyses did not show an increased incidence of SOS after HSCT in patients treated with GO [6,7]. This may be due to the use of lower, fractionated doses of GO, longer intervals between GO and HSCT, improved patient selection, and better recognition and treatment of SOS.

In this patient, GO was administered at 3 mg/m² on days 1, 4, and 7 of induction therapy, following the ALFA-0701 trial [8]. However, despite this adjusted dosing, the patient still developed very severe SOS. This demonstrates that the risk of SOS associated with GO remains an important consideration in its use and underscores the need for careful patient selection and monitoring. Recent studies suggest that even fewer doses of GO may offer survival benefits and improve MRD negativity rates [9]. Therefore, further reducing the dose to one or two administrations of 3 mg/m² may be considered for patients at risk of developing SOS.

Other than treatment with GO (three fractionated doses of 3 mg/m^2^) this patient had no major risk factors for developing SOS. However, our patient was cytomegalovirus (CMV) seropositive, which has been reported as a minor risk factor for the development of SOS [4]. In a large retrospective cohort study of patients who underwent myeloablative HSCT, positive CMV serology in the patient or donor was independently associated with SOS development [10].

Despite the relatively low predetermined risk, this patient developed a very severe SOS, which might indicate that additional risk factors for the development of SOS are not yet identified, in particular in patients treated with GO and outside the scope of HSCT.

Given the high mortality rate of very severe SOS, treatment should be started as soon as possible. Defibrotide is the only agent with proven efficacy for the treatment of (very) severe SOS at a recommended dose of 25 mg/kg/day. The DEFIFrance study reported infection (17%) and hemorrhage (16%) as the most frequent side effects in patients with severe/very severe SOS post-HSCT, which is consistent with other studies [11].

Before the introduction of defibrotide, treatment with high-dose methylprednisolone was often used. However, little supporting data is available, mostly from retrospective studies and studies with limited numbers of patients. Furthermore, high-dose corticosteroids might increase the risk of infectious complications. Therefore, the British Committee for Standards in Haematology and the British Society for Blood and Bone Marrow Transplantation state that the use of steroids should only be considered in (very) severe SOS [12]. Moreover, Gloude et al. showed in a single-center, retrospective study of pediatric patients that the combination of high-dose steroids and defibrotide may be superior to defibrotide in monotherapy [3]. This combination therapy needs further investigation.

SOS primarily affects the small veins and sinusoids in the liver, leading to obstruction and damage. This obstruction results in the development of portal hypertension. TIPS is a percutaneous, minimally invasive technique used to rapidly and effectively decrease portal hypertension and its associated complications. This technique is not yet commonly used for the treatment of SOS.

In a recent retrospective analysis of 185 patients with haploidentical HSCT, 17 patients were diagnosed with SOS. Among them, 13 patients developed (very) severe SOS. All these patients, except one, were treated with defibrotide. Seven patients with very severe SOS also received TIPS, with a median time from SOS diagnosis to TIPS insertion of three days. Following TIPS, almost all patients showed clinical improvement, including amelioration of kidney function and liver tests, with a 100-day overall survival rate of 100% [13]. Undelayed placement of TIPS appears to be crucial. The use of TIPS in SOS was first described in a cohort of 10 patients in 2000, where it successfully reduced portal hypertension [14]. However, in that study, TIPS was inserted relatively late, with a mean of 35 ± 53 days after SOS diagnosis. Consequently, 90% of those patients died soon after the procedure due to their SOS. This earlier study did not include treatment with defibrotide, which likely contributed significantly to the improved survival rates observed in the more recent studies. Nevertheless, TIPS seems to have a role in selected patients with very severe and rapidly progressing SOS.

## Conclusions

In this case report, we present a patient with very severe SOS after treatment with GO, in the absence of other known risk factors. This case underscores the critical importance of early recognition and aggressive management of SOS in patients undergoing high-risk treatments such as GO. We report a unique and successful combination strategy of immediate mechanic reduction of portal hypertension with TIPS and drug-mediated inhibition of microvascular thrombosis leading to rapid clinical improvement. Furthermore, this case also suggests that further research is needed to refine treatment protocols and identify additional risk factors for SOS, ensuring better prevention and management strategies for future patients.
